# ir-HSP: Improved Recognition of Heat Shock Proteins, Their Families and Sub-types Based On *g*-Spaced Di-peptide Features and Support Vector Machine

**DOI:** 10.3389/fgene.2017.00235

**Published:** 2018-01-11

**Authors:** Prabina K. Meher, Tanmaya K. Sahu, Shachi Gahoi, Atmakuri R. Rao

**Affiliations:** ^1^Division of Statistical Genetics, ICAR-Indian Agricultural Statistics Research Institute, New Delhi, India; ^2^Centre for Agricultural Bioinformatics, ICAR-Indian Agricultural Statistics Research Institute, New Delhi, India

**Keywords:** molecular chaperones, heat shock, protein folding, machine learning, di-peptide composition, DnaJ proteins

## Abstract

Heat shock proteins (HSPs) play a pivotal role in cell growth and variability. Since conventional approaches are expensive and voluminous protein sequence information is available in the post-genomic era, development of an automated and accurate computational tool is highly desirable for prediction of HSPs, their families and sub-types. Thus, we propose a computational approach for reliable prediction of all these components in a single framework and with higher accuracy as well. The proposed approach achieved an overall accuracy of ~84% in predicting HSPs, ~97% in predicting six different families of HSPs, and ~94% in predicting four types of DnaJ proteins, with bench mark datasets. The developed approach also achieved higher accuracy as compared to most of the existing approaches. For easy prediction of HSPs by experimental scientists, a user friendly web server ir-HSP is made freely accessible at http://cabgrid.res.in:8080/ir-hsp. The ir-HSP was further evaluated for proteome-wide identification of HSPs by using proteome datasets of eight different species, and ~50% of the predicted HSPs in each species were found to be annotated with InterPro HSP families/domains. Thus, the developed computational method is expected to supplement the currently available approaches for prediction of HSPs, to the extent of their families and sub-types.

## Introduction

In the course of constant interaction between life and environment, cell experiences different environmental stresses that slow down its original function including DNA, RNA, and protein synthesis (Csermely and Yahara, 2005). Further, almost all these stresses induce a group of proteins called as heat shock proteins (HSPs) that are found almost in all living organisms (Al-Whaibi, 2011). HSPs are considered as a major group of molecular chaperones assisting in proper folding of partially folded or denatured proteins, organization of correct protein conformation, and prevention of irreversible aggregation of damaged proteins (Hubbard and Sander, 1991; Zeng et al., 2004; Poulain et al., 2010). Six major families of HSPs viz., HSP20, HSP40, HSP60, HSP70, HSP90, and HSP100 have been reported based on molecular weight and functions (Ratheesh et al., 2012; Feng et al., 2013). Besides chaperonin activities, HSPs are also known to be involved in other functions like modulation of their synthesis (Blaszczak et al., 1999), participation in signal transduction pathways (Louvion et al., 1998), RNA processing (Ruggero et al., 1998) etc. Furthermore, HSPs play vital role in maintaining the overall cellular protein homeostasis (Mallouk et al., 1999; Tytell and Hooper, 2001). Due to broad range of functions of HSPs, their dysfunction causes many serious disorders. In particular, aggregation of misfolded proteins causes many neurodegenerative diseases including Alzheimer's and Parkinson's disease (Hamos et al., 1991; Wu et al., 2004; Adachi et al., 2009; Arawaka et al., 2010; Sajjad et al., 2010; Maiti et al., 2014; Paul and Mahanta, 2014; Lackie et al., 2017), cardiovascular disease (Pockley, 2002), and cancer (Goldstein and Li, 2009). The HSPs can also be used as therapeutic targets for cancer treatment (Chatterjee and Burns, 2017; Saini and Sharma, 2017; Wu et al., 2017), diagnosis of neurodegenerative disorders (Kampinga and Bergink, 2016), and preventing the onset and progression of atrial fibrillation (Hu et al., 2017). Owing to the wide range of activities of HSPs, identification of HSPs, and categorizing them into different families is an important and challenging problem (Feng et al., 2013; Ahmad et al., 2015).

The advancement of relatively cheaper sequencing technologies has witnessed a huge volume of protein sequences that are added to the public databases (Feng et al., 2013; Ahmad et al., 2015; Kumar et al., 2016). Due to lack of experimentally validated structures in the databases, resource intensive traditional method like nuclear magnetic resonance (NMR) has become inappropriate for identifying HSP families in large protein datasets (Redfield, 2004; Lange et al., 2012; Nasedkin et al., 2015). Thus, the development of computational method for identifying HSPs and their families is essential due to their inexpensive and high throughput nature. To this end, some computational methods i.e., iHSP-PseRAAAC (Feng et al., 2013), JPred (Feng et al., 2014), JPPRED (Zhang et al., 2015), and PredHSP (Kumar et al., 2016) have already been developed in the recent past. In iHSP-PseRAAAC, support vector machine (SVM) was used for predicting six different families of HSPs, based on reduced alphabet amino acid composition (AAC) features of protein sequences. The JPred was developed for prediction of four types of HSP40 (DnaJ) proteins (Type-I, Type-II, Type-III, and Type-IV) based on composition of clustered amino acids and SVM predictor. In another approach, Ahmad et al. (2015) employed different supervised learning techniques viz., k-nearest neighbor, probabilistic neural network, SVM, and artificial neural network for prediction of six different families of HSPs as well as four different types of DnaJ proteins, based on different compositional features viz., pseudo AAC, split AAC, and di-peptide compositions (DPC). The JPPRED employed a more complex set of features for predicting different DnaJ proteins. Keeping in mind the fact that a protein sequence should be predicted first as HSP before being predicted to its family, PredHSP was developed based on DPC using SVM. It identifies HSPs in the first stage and classifies them into different families in the second stage. Each of the above mentioned approaches has their own advantages, and contributed in generating knowledge for predicting HSPs. Though reasonable results have been achieved in identifying HSP families, still there is a room for improvement. In particular, there is a need to improve the accuracy of prediction of HSPs and non-HSPs. Moreover, none of the above mentioned tools were designed to predict all the three components viz., HSPs, six families of HSPs, and four types of DnaJ proteins in a single framework. Thus, the development of a new computational approach is required for the prediction of all these three components reliably, and in a single framework as well.

Taking above prospects into consideration, we made an attempt in this study to develop a novel computational method for predicting all the three components in a single framework. In the proposed approach, G-spaced di-peptide compositions were used as input features and SVM as the prediction machine. The proposed approach achieved higher accuracy than most of the existing approaches in predicting HSPs, six families of HSPs and four types of DnaJ proteins, while compared using benchmark datasets. Besides, an online prediction server has also been developed to help enable the researchers, scientists and other stakeholders in predicting HSP families and their sub-types with higher accuracy.

## Materials and methods

As stated in many recently published articles (Chen et al., 2016; Jia et al., 2016c; Liu B. et al., 2016; Liu Z. et al., 2016; Qiu et al., 2016; Meher et al., 2017), five steps should be followed to set up a sequence-derived-features based statistical predictor. The steps are as follows:

- Build standard training and test datasets to effectively train and test the predictor.
- Map the input biological sequences into such numeric feature vectors which can truly reflect their inherent association with the target.
- Develop an efficient prediction algorithm.
- Properly perform the cross-validation tests.
- Develop an online prediction server, which is freely accessible to the users.

In the following sub-sections, we have described these steps one-by-one.

### Dataset

We considered the same dataset which was used to develop PredHSP. This dataset contains 2,225 true HSP and 10,000 non-HSP sequences. The true HSP dataset was actually constructed by Feng et al. (2013) to develop iHSP-PseRAAAC, where the sequences were originally collected from HSPIR database (Ratheesh et al., 2012). Though HSPIR contains >9,900 sequences belonging to 277 genomes of both prokaryotes and eukaryotes, the true HSP dataset was constructed after removing the sequences with ≥40% pair-wise sequence identity in each family of HSP, to reduce homologous bias and redundancy. Further, the non-HSP dataset consisting of 10,000 sequences was created first time in PredHSP, where the sequences were randomly drawn from Swiss-Prot (http://web.expasy.org/docs/swiss-prot_guideline.html) based on the criterion that no two sequences are homologous. After removing the sequences with non-standard residues (residues other than 20 amino acids), a final dataset consisting of 2,181 HSPs (354 HSP20, 1,257 HSP40, 159 HSP60, 278 HSP70, 52 HSP90, and 81 HSP100) and 9,965 non-HSPs was prepared (Table 1).

**Table 1:** Summary of the positive and negative datasets.

**Class**	**Dataset**	**Description**	#Sequence^*^
Positive	HSP20	sHSP	354
	HSP40	DnaJ-class proteins	1,257
	HSP60	GroEL/ES or chaperonin	159
	HSP70	DnaK/chaperones	278
	HSP90	HptG or Chaperonin	52
	HSP100	Clp	81
Negative	non-HSP	—	9,965

* *Sequences obtained after removing non-standard residues*.

### Construction of balanced dataset

The final dataset (2,181 HSPs and 9,965 non-HSPs) is highly imbalanced, because the number of sequences in non-HSP dataset are much higher than that of HSP dataset. By using the highly imbalanced dataset to train the prediction model, the results may get biased toward the class having larger number of sequences i.e., major class (Chou, 2013; Chen et al., 2015; Liu Z. et al., 2015; Xiao et al., 2015; Jia et al., 2016b; Liu B. et al., 2017). In order to reduce the biasness, balanced datasets having approximately same number of HSP and non-HSP sequences were constructed for classification of HSPs and non-HSPs. More clearly, balanced datasets consisting of 2,180 HSPs and 2,180 non-HSPs were prepared for classification of HSPs and non-HSPs, which were randomly drawn from 2,181 HSPs and 9,965 non-HSPs, respectively. Classifications were also made among different families of HSPs where a particular family is considered as the positive set and the remaining families together as negative set. Moreover, performances of prediction models were assessed using leave-one-out cross-validation (LOOCV) technique as similar to the earlier studies (Feng et al., 2013; Ahmad et al., 2015; Kumar et al., 2016).

### Feature generation

Sequence-derived features viz., AAC and DPC were previously used by Kumar et al. (2016) where the accuracy under DPC feature was found to be higher than that of AAC. The reason behind this could be the local ordering of amino acids that are not accounted in AAC. On the other hand, the DPC not only encapsulates the local ordering of amino acids but also the global information of each protein sequence (Bhasin and Raghava, 2004; Ding et al., 2004). Keeping this in mind, four kinds of DPC i.e., 0-spaced, 1-spaced, 2-spaced, and 3-spaced were used, which are nothing but the frequencies of all pairs of amino acids conditioned with 0, 1, 2, and 3 skips, respectively (Govindan and Nair, 2011). Besides, all possible combinations of 0-, 1-, 2-, and 3-gap (spaced) amino acid pair compositions (GPC) were also used as features. Since, composition-transition-distribution (CTD), autocorrelation function (ACF), and pseudo-AAC (PAAC) features also take into account the local ordering of amino acids as similar to GPC, they were considered as features. For computing these features, *BioSeqClass* package (Hong, 2016) of R-software (R Development Core Team, 2012) was used. A brief description about the computation of GPC, PAAC, CTD, and ACF features is provided below.

#### G-spaced amino acid pair composition (GPC)

Each kind of GPC gives 400 descriptors, which can be defined as $f_{G}\left(i,\;j\right)=\frac{D_{G}\left(i,\;j\right)}{N-G-1}\;\left(i,\;j=1,2,\ldots ,20;\;G=0,\;1,2,3\right)$, where *D*_*G*_(*i, j*) is the number of amino acid pairs represented by amino acid *i* and *j* with *G*-gap, *f*_*G*_(*i, j*) represents the frequency of occurrence and *N* is the length of sequence.

#### Pseudo amino acid composition (PAAC)

The PAAC was first time used by Chou (2001) for the prediction of protein sub-cellular localization. Unlike the discrete AAC, the effects of sequence ordering are taken into consideration in PAAC. This feature has been verified effectively in many protein-related classifications (Wang et al., 2010). Based on the PAAC features, each protein sequence can be mapped onto a (20+d)-dimensional numeric feature vector for d-tier correlation factor. In the current study, 1^st^-tier correlation was only considered by which each sequence was converted into a numeric vector of 21 elements. Though more details can be found from the studies of Chou (2005, 2009), a brief description about computing the PAAC features is as follows:

Let ψ_1_ψ_2_ψ_3_ … ψ_*L*−2_ψ_*L*−1_ψ_*L*_ be a protein sequence of *L* amino acids long. Then, the ordering of amino acids in the sequence can be represented by a set of discrete correlation factors ρ_1_, ρ_2_, …, ρ_*d*_, where

$$\begin{array}{l} \rho_{j}=\frac{1}{L-j}\overset{L-j}{\underset{i\;=\;1}{\sum }}\Phi \left(\psi_{i},\psi_{i+j}\right);j\;=\;1,2,\ldots ,d\left(<L\right). \end{array}$$

The ρ_1_, ρ_2_, …, ρ_*d*_ are called the 1^st^, 2^nd^, …, d^th^ tier correlation factors, respectively. The correlation function Φ(ψ_*i*_, ψ_*i*+*j*_) is given by ${\left[\Theta \left(\psi_{i}-\psi_{i+j}\right)\right]}^{2}$, where Θ(ψ_*i*_) is the transformed feature value of amino acid ψ_*i*_. The value of Θ(ψ_*i*_) can be computed from the original feature value Θ_*o*_(ψ_*i*_) as follows:

$$\begin{array}{l} \Theta (\psi_{i})=\frac{\Theta_{o}(\psi_{i})-\overset{20}{\underset{i\;=\;1}{\sum^{\;}}}\frac{\Theta_{o}(\psi_{i})}{20}}{\sqrt{\frac{\overset{20}{\underset{i\;=\;1}{\sum^{\;}}}{\left[\Theta_{o}(\psi_{i})-\overset{20}{\underset{i\;=\;1}{\sum^{\;}}}\frac{\Theta_{o}(\psi_{i})}{20}\right]}^{2}}{20}}}. \end{array}$$

Thus, the PAAC of a protein can be represented by a (20+*d*)-dimensional vector as [θ_1_, θ_2_, …, θ_20_, θ_21_, …, θ_20+d_]′, where θ_*x*_ is represented as

$$\begin{array}{l} \theta_{x}=\{\begin{array}{l} \frac{f_{x}}{\sum_{i\;=\;1}^{20}f_{i}+w\sum_{j\;=\;1}^{d}\rho_{j}}\;1\leq x\leq 20\\ \frac{w\rho_{x}-20}{\sum_{i\;=\;1}^{20}f_{i}+w\sum_{j\;=\;1}^{d}\rho_{j}},\;21\leq x\leq 20+d \end{array}\;, \end{array}$$

where *f*_*x*_ represents the occurrence of frequencies for the 20 amino acids in the protein sequence, ρ_*j*_ represents the *j*^th^ tier sequence correlation factor and *w* represents the weight for the sequence-order effect.

#### Composition-transition-distribution (CTD)

Dubchak et al. (1995) introduced the CTD feature for the prediction of protein folding classes. Since its introduction, it has been widely used in many functional and structural related studies of proteins (Cai et al., 2003; Govindan and Nair, 2011). In CTD feature, composition (C) stands for the composition of amino acids, transition (T) represents the percent frequency with which residues of certain characteristics are followed by other amino acids, and distribution (D) determines the sequence length within which the first, 25, 50, 75, and 100% of the amino acids of certain characteristics are placed. With the CTD feature, each protein sequence of length *L* was mapped into a numeric vector of length *L* + *{L* ^*^ *(L*−*1)/2}* + *(L* ^*^ *5)*.

#### Autocorrelation function (ACF)

Features based on ACF take into consideration the dependencies between the sequence features at each location. ACF-based features are computed by taking into account the distribution of amino acid properties along the sequence. In this study, ACF features were computed based on all the 531 amino acid indices available in AAindex database (Kawashima and Kanehisa, 2000). With ACF feature encoding, each sequence was transformed into a 531^*^n-dimensional numeric feature vector, for *n*^th^ order autocorrelation. Here, we considered the 1^st^ and 2^nd^ order autocorrelation only, because the number of features will increase geometrically with increase in the order.

### Support vector machine (SVM)

The SVM supervised learning technique (Cortes and Vapnik, 1995) has been extensively used in the area of computational biology and bioinformatics (Chou and Cai, 2002; Chen and Lin, 2010; Lin and Ding, 2011; Xiao et al., 2012; Chen et al., 2013). In the context of predicting HSPs, SVM has already been used in earlier studies (Feng et al., 2013, 2014; Ahmad et al., 2015; Zhang et al., 2015; Kumar et al., 2016). The kernel functions play vital role as far as the predictive ability of SVM is concerned. Using the kernel function, the input dataset is transformed into a high-dimensional feature space in which the observations of different classes are linearly separable by optimal separating hyper plane. We also employed SVM for prediction purpose in this study. Based on a sample dataset of 1,000 HSPs and 1,000 non-HSPs, all the four basic kernels (Linear, Polynomial, Radial, and Sigmoid) with default parameters setting were initially used to assess the prediction accuracy. Then, the model with the best fitted kernel (having highest accuracy) was chosen and used in the subsequent analysis. The *svm* function available in *e1071* package (Dimitriadou et al., 2012) of R-software was used for implementing SVM model.

### Evaluating the performance

Cross-validation is an essential tool in machine learning and statistics. This procedure estimates the expected error of a learning algorithm by running training and testing procedures repeatedly on different partitions of the dataset (Geras and Sutton, 2013). Here, five-fold cross-validation procedure was adopted for evaluating the performance of the developed approach. In this procedure, the dataset was partitioned into five sets randomly, where in each set almost same number of HSPs and non-HSPs were present. Four out of five sets were used to train the prediction model and the remaining one set was used for validation. Each set was used once for validation and thus the whole process was repeated five times. The performance of the method was measured by taking average over the five sets. We considered the evaluation metrics, *viz*., sensitivity, specificity, accuracy, precision, and Matthew's correlation coefficient (MCC) to evaluate the performance of the proposed approach because these measures have been widely accepted by researchers (Guo et al., 2014; Lin et al., 2014; Liu B. et al., 2014, 2016; Jia et al., 2016a,d; Liu et al., 2016; Meher et al., 2017) for assessing the performance of statistical predictor. The above mentioned performance metrics are defined as follows:

$$\begin{array}{l} Sensitivity=\frac{true\;positive\;\left(tp\right)}{true\;positive\;\left(tp\right)+false\;negative\;\left(fn\right)};\\ Specificity=\frac{true\;negative\;\left(tn\right)}{tn+false\;positive\;\left(fp\right)};\\ Acccuracy=\frac{tp+tn}{tp+fn+tn+fp};Precision=\frac{tp}{tp+fp};\\ MCC=\frac{\left(tp\times tn\right)-\left(fp\times fn\right)}{\sqrt{\left(tp+fn\right)\times \left(tp+fp\right)\times \left(tn+fn\right)\times \left(tn+fp\right)}}, \end{array}$$

where *tp, tn, fp*, and *fn* represent the number of HSPs correctly classified, non-HSPs correctly classified, non-HSPs misclassified as HSPs and HSPs misclassified as non-HSPs, respectively. As receiver operating characteristics (ROC) is also a widely used measure (Baten et al., 2006), we further used area under ROC curve (AUC-ROC) (Fawcett, 2006; Davis and Goadrich, 2013) to evaluate the prediction accuracy of the proposed approach. Furthermore, ROC is independent of class distribution and precision-recall is a better measure over ROC under imbalanced situation. Thus, areas under precision-recall curve (AUC-PR) were used for comparing the performance of the developed approach with the existing methods.

### Comparison with existing methods

PredHSP is the only tool available in literature for classification of HSP and non-HSP proteins. Thus, comparison was made between the performances of PredHSP and the proposed approach by using two independent datasets. The first independent dataset contains 96 human HSPs collected from HUGO Gene Nomenclature Committee (HGNC) database and the second dataset comprises of 55 rice HSPs, where 31 HSPs (14 HSP20, 4 HSP60, 7 HSP70, 3 HSP90, and 3 HSP100) were obtained from Wang et al. (2014) and 24 HSP70 were obtained from Sarkar et al. (2013). We used these datasets to compare our developed approach with the PredHSP, as the same datasets have been used to evaluate the performance of PredHSP. Besides, we have also prepared a non-HSP dataset consisting of 5,000 sequences that were randomly drawn from UniProt (http://www.uniprot.org/), where none of the sequences has >40% pair-wise sequence identity to any other sequences in the dataset. We constructed this independent negative dataset to evaluate the performance of the PredHSP as well as to compare with that of proposed approach, because the PredHSP has not been evaluated with any independent negative dataset. Further, the classification accuracy of the developed approach was compared against the existing methods viz., PredHSP, iHSP-PseRAAAC, and Ahmad et al. (2015) approach with respect to classification of different families of HSPs. The performances were compared using 354 HSP20, 1,257 HSP40, 159 HSP60, 278 HSP70, 52 HSP90, and 81 HSP100 sequences, because the same datasets have been used to evaluate the performance of PredHSP, iHSP-PseRAAAC, and Ahmad et al. (2015) approach for classifying different families of HSPs. For classification among different families of HSPs, LOOCV technique was employed to assess the performances.

### Prediction of DnaJ protein types

Besides classifying different families of HSPs, classifications were also made among four different types of J-proteins (Type-1, Type-II, Type-III, and Type-IV). The sequences of J-proteins were obtained from an earlier study (Feng et al., 2014), accessible at http://cabgrid.res.in:8080/ir-hsp/dataset.html. These J-proteins datasets, which were originally derived from HSPIR database, were prepared after removing the sequences with non-standard residues as well as the sequences having >40% pair-wise sequence identities. The constructed dataset comprises of 63 Type-I, 53 Type-II, 1,107 Type-III, and 22 Type-IV sequences. Since the number of sequences are small in different types (except Type III), LOOCV was adopted for assessing the performance. These datasets have been used to evaluate the performances of JPred and JPPRED, for classification of four types of DnaJ proteins. Therefore, we have also used the same datasets to evaluate the proposed computational approach as well as to compare with the above mentioned approaches. As the datasets are highly imbalanced, AUC-PR was also used along with the other metrics for comparing the performances.

### Performance evaluation with interpro dataset

Since, the positive independent datasets used to evaluate the performances of PredHSP and the proposed approach are very small (96 human HSPs and 55 rice HSPs, as mentioned in section Comparison with Existing Methods), the predictive abilities of the developed computational method and PredHSP were also assessed using HSPs of different families, which were collected from InterPro database (https://www.ebi.ac.uk/interpro/). The number of sequences in different families/domains, obtained after removing the non-standard amino acids, are provided in Table 2. We did not consider HSP100 because no match was found for the keyword HSP100 in InterPro.

**Table 2:** Number of HSP sequences collected from InterPro, corresponding to different HSP families.

**HSP family**	**InterPro ID**	**Description**	**#Sequence**
HSP 20	IPR031107	Small heat shock protein family	12,642
HSP 40	IPR001305	Heat shock protein DnaJ, Cysteine rich domain	22,900
HSP 60	IPR001844	Chaperonin Cpn60	18,801
HSP 70	IPR012725	Chaperone DnaK	14,366
HSP 90	IPR001404	Heat shock protein HSP90 family	15,233

### Proteome-wide identification

The proposed approach was also employed for proteome-wide identification of HSPs and their families. Since HSPs are present in all the three domains of life, we considered eight different proteomes belonging to archaea (*Methanothermobacter thermautotrophicus*), prokaryotes (*Mycobacterium tuberculosis* and *Escherichia coli*), and eukaryotes (*Arabidopsis thaliana, Saccharomyces cerevisiae, Drosophila melanogaster, Oryza sativa*, and *Caenorhabditis elegans*). The total number of proteins collected are 1,857, 4,187, 3,873, 6,479, 30,036, 37,228, 25,878, 20,249 for *M. thermautotrophicus, E. coli, M. tuberculosis, S. cerevisiae, A. thaliana, O. sativa, C. elegans*, and *D. melanogaster*, respectively.

### Development of prediction server

To augment the practical applicability of the developed approach as well as to make use of the proposed approach convenient for the experimental scientists, a web server was also designed and hosted for the prediction of HSPs, their families and sub-types of DnaJ proteins. The server was developed using hypertext mark-up language (HTML) and hypertext pre-processor (PHP), where an in-house R-script was executed in the backend upon submitting protein sequences in single letter code format. The user can submit one or more protein sequences in FASTA format where each sequence should contain only standard amino acid residues.

## Results

### Analysis of kernels and features

**Figure 1:**
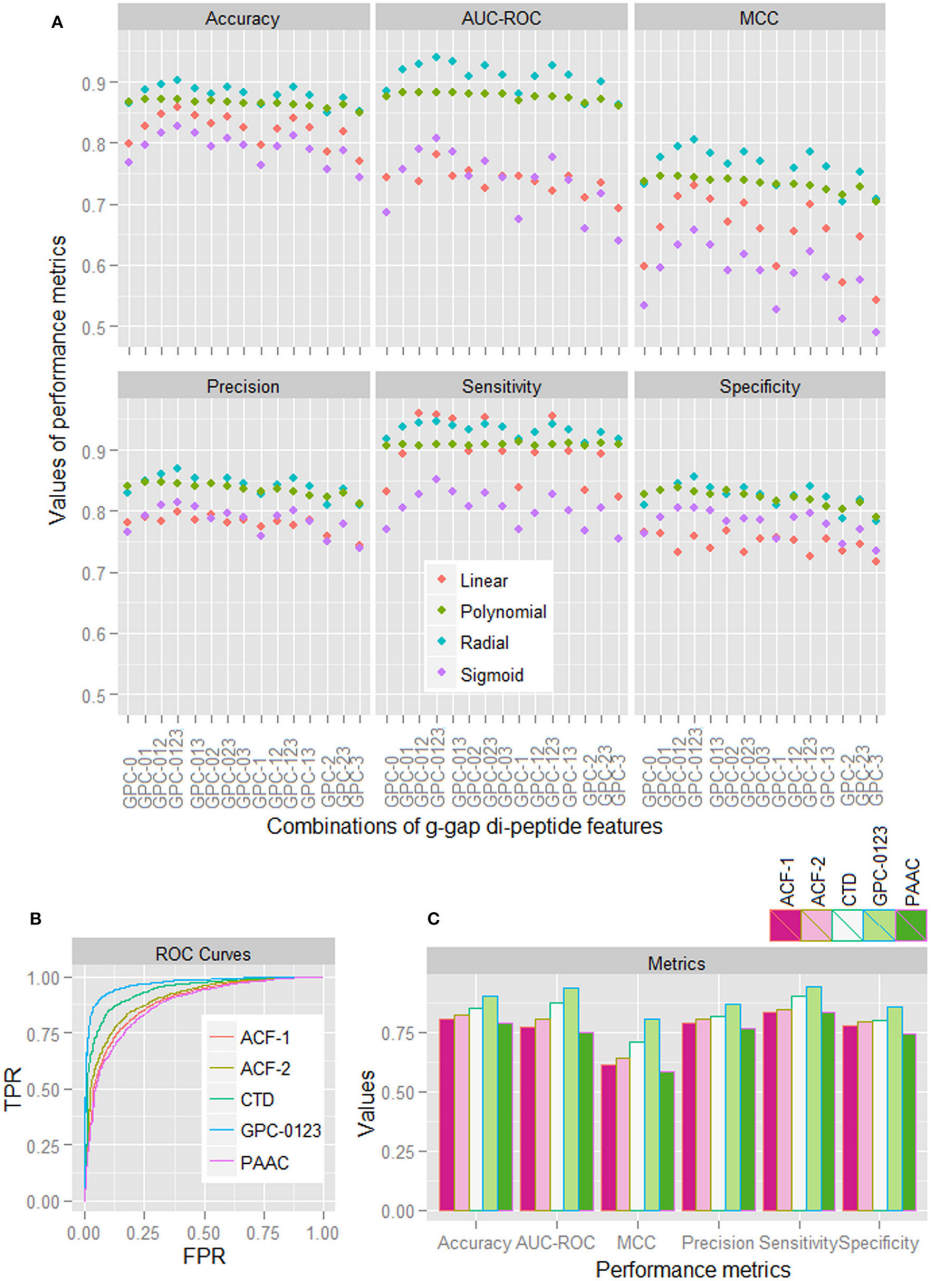
**(A)** Performance metrics of SVM for four different kernels, under different combinations of 0-, 1-, 2-, and 3-spaced base pair features. **(B)** ROC curves for SVM with RBF kernel, under different feature sets i.e., ACF-1, ACF-2, CTD, GPC-0123, and PAAC. **(C)** Values of different performance metrics for SVM with RBF kernel under different feature sets. It can be seen that the values of performance metrics for SVM with RBF kernel under GPC-0123 feature set are higher than that of other feature sets.

Based on the sample dataset of 1,000 HSPs and 1,000 non-HSPs, performance metrics for different combinations of GPC features (computed by taking average over five-folds) for all the four kernels are shown in Figure 1A. With some exceptions in sensitivity, it is observed that the performance metrics for the radial basis function (RBF) kernel are higher than that of other kernels. It is further observed that the performance metrics are higher for the combined 0-, 1-, 2-, and 3-gap amino acid pair features (GPC-0123), irrespective of the kernels used. From ROC curves of different features (Figure 1B), it is also seen that the area covered under ROC curve of GPC-0123 feature is higher than that of other feature sets i.e., PAAC, CTD, ACF of 1^st^ order (ACF-1), and ACF of 2^nd^ order (ACF-2). In addition, sensitivity, specificity, accuracy, and MCC are also observed to be higher for the GPC-0123 feature set (Figure 1C). Furthermore, it can be observed that the performance metrics under GPC-0123 feature set are much higher than that of DPC feature i.e., GPC-0 (Figure 1A), which is adopted in PredHSP for the prediction of HSPs and their families.

### Feature selection analysis

**Figure 2:**
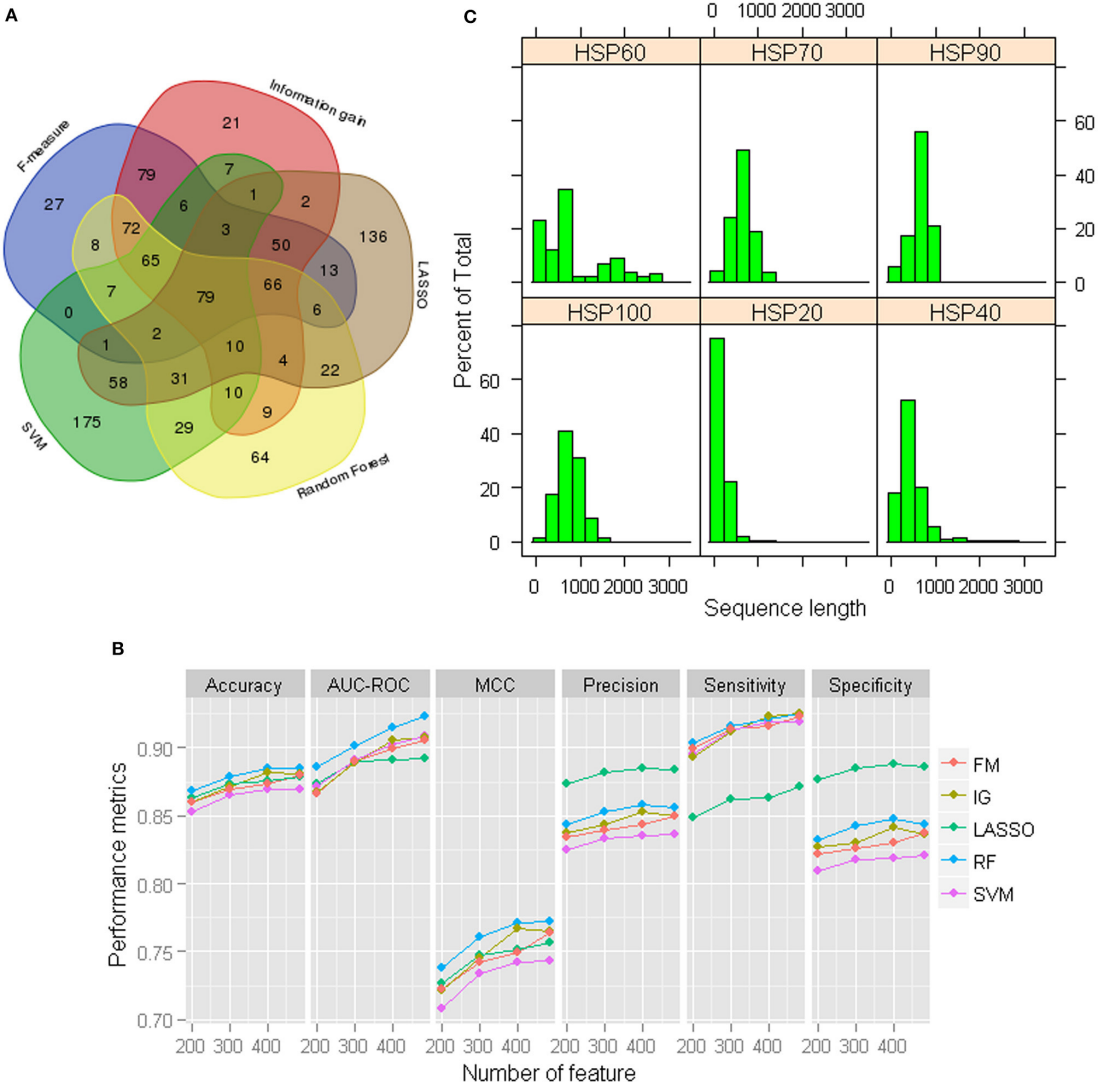
**(A)** Venn diagram of the selected 484 features under each feature selection technique. It can be seen that a large number of features selected under LASSO and SVM are not common to the features selected under other techniques. On the other hand, most of the features selected through F-measure and Information gain are among those selected under other three techniques. **(B)** Performance metrics for SVM with RBF kernel with 484 selected GPC-0123 features under each feature selection technique. It can be observed that values of performance metrics are higher for RF-based selected 484 GPC-0123 features. **(C)** Distribution of length of sequences for each family of HSPs.

Although the prediction accuracies are observed to be higher for GPC-0123 feature set (Figure 1), the number of features in GPC-0123 are large (1,600) and prediction analysis by using such a large number of features may take longer time. Thus, we employed five different feature selection techniques viz., F-measure (FM) (Golub et al., 1999), Information gain (IG) (Alhaj et al., 2016), LASSO (Tibshirani, 1996), Random Forest (Breiman, 2001), and SVM (Cortes and Vapnik, 1995) to select important features. The criteria for selecting important features under each technique are provided in Data Sheet 1. Since 484 features are observed with non-zero coefficients under LASSO, same number of features are also selected under other techniques. Among 484 selected features, it is observed that most of the features selected under IG and FM are among those selected through other three selection techniques (Figure 2A). On the contrary, large number of features selected under SVM and LASSO are not among the features selected through other three techniques. Based on the selected features under each technique, performance of SVM was also assessed using the sample dataset of 1,000 HSPs and 1,000 non-HSPs. Except specificity and precision, higher values of performance metrics are observed under 484 RF-based selected features (Figure 2B). Thus, the 484 RF-based selected features are considered in subsequent analysis.

### Cross validation analysis

Using 484 RF-based selected features, prediction was made for 2,180 HSPs and 2,180 non-HSPs that were randomly drawn from 2,181 HSPs and 9,965 non-HSPs, respectively. Moreover, to assess the consistency of the proposed approach, prediction was made over 100 such samples (where each sample consists of 2,180 HSPs and 2,180 non-HSPs) that were randomly drawn from the available HSP and non-HSP sequences. Using the same datasets, performance of PredHSP (DPC as features and SVM as classifier) was also evaluated. Performance metrics averaged over five-folds as well as 100 sample sets are given in Table 3. From the table, it is observed that the specificities are higher than the sensitivities. The proposed approach achieved ~84% overall accuracy, which is 2% higher than that of PredHSP (~82%). In terms of all the performance metrics, proposed approach is also observed to achieve higher accuracy than that of PredHSP. Except MCC, values of other performance metrics for the proposed approach are observed >80%. On the other hand, except specificity and precision, values of other performance metrics are <80% for PredHSP. Besides, the performance metrics of the proposed approach are also seen to be more stable (less standard error) as compared to that of PredHSP.

**Table 3:** Performance metrics for the proposed approach with respect to classification of HSP and non-HSP sequences.

**Method**	**Sensitivity**	**Specificity**	**Accuracy**	**Precision**	**MCC**	**AUC-ROC**	**AUC-PR**
Proposed	0.8262 (±0.0049)	0.8578 (±0.0047)	0.8420 (±0.0037)	0.8532 (±0.0043)	0.6844 (±0.0074)	0.8401 (±0.0046)	0.8567 (±0.0041)
PredHSP	0.7788 (±0.0052)	0.8190 (±0.0051)	0.7989 (±0.0041)	0.8114 (±0.0046)	0.5983 (±0.0080)	0.7558 (±0.0063)	0.7712 (±0.0071)

*Value inside bracket indicates standard error*.

### Family-wise performance analysis

Based on the 484 selected features, classifications were further made among different families of HSPs by following LOOCV technique. The values of different performance metrics are given in Table 4. Overall accuracies of >96% are observed for all the HSP families. It is also observed that the sensitivity, specificity and MCC are higher for HSP40 as compared to the other families of HSPs, and this may be due to the large number of sequences in HSP40 that lead to a well fitted prediction model. On the other hand, it is seen that the performance metrics (sensitivity, precision, MCC) are low for HSP60, and this may be due to the larger variability in the sequence length as compared to other families (Figure 2C) as well as the number of sequences in that family is 159 by which the model was not fitted well. Similarly, the sensitivity for HSP90 is also low (75%), and the possible reason for this may be that the number of sequences is less (52). Since the datasets were highly imbalanced, specificities are observed to be higher than the sensitivity. In terms of AUC-ROC and AUC-PR, accuracies are observed to be higher for HSP100 followed by HSP40 and HSP20. On the other hand, lowest values of AUC-ROC and AUC-PR are seen for HSP90 (Table 4).

**Table 4:** Performance metrics for the proposed approach with regard to classification of different families of HSPs.

**HSP family**	**Sensitivity**	**Specificity**	**Accuracy**	**Precision**	**MCC**	**AUC-ROC**	**AUC-PR**
HSP20	0.9463	0.9661	0.9628	0.8438	0.8718	0.9835	0.4860
HSP40	0.9745	0.9513	0.9647	0.9645	0.9276	0.9868	0.4884
HSP60	0.6792	0.9886	0.9661	0.8244	0.7307	0.9480	0.4516
HSP70	0.8849	0.9884	0.9752	0.9179	0.8871	0.9547	0.4633
HSP90	0.7500	0.9976	0.9917	0.8863	0.8112	0.8942	0.4277
HSP100	0.8889	0.9957	0.9917	0.8889	0.8846	0.9937	0.4935

### Comparative analysis of family-wise prediction

**Figure 3:**
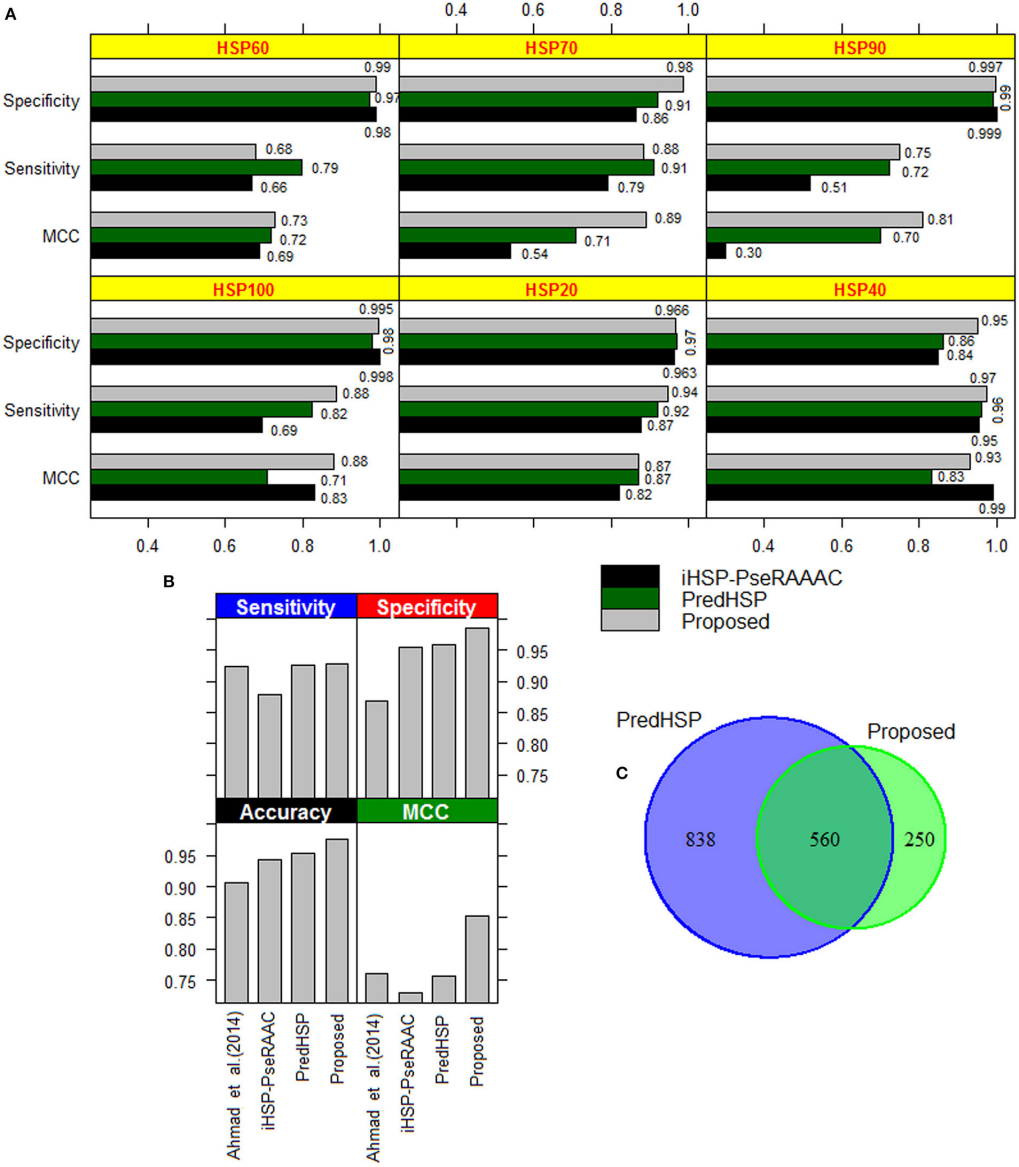
**(A)** Bar diagrams of performance metrics for the proposed approach, PredHSP and iHSP-PseRAAAC with respect to classification of different families of HSPs. With some exceptions (sensitivity in HSP60 and HSP70; MCC in HSP40), proposed approach is seen to achieve higher accuracy than that of other two approaches. **(B)** Bar diagrams of weighted accuracies for the above mentioned three approaches along with the Ahmad et al. (2015) approach. It can be seen that the weighted accuracies are higher for the proposed approach followed by PredHSP. **(C)** Venn diagram showing the number of HSPs predicted common (i.e., 560) to the proposed approach and PredHSP, for the negative dataset of 5,000 protein sequences.

The performances of the developed approach, PredHSP and iHSP-PseRAAAC were compared in respect of classification of families of HSPs. Since family-wise accuracy is not available for Ahmad et al. (2015) approach, weighted average accuracies were also compared. Family-wise accuracies are shown in Figure 3A, and the weighted average accuracies are shown in Figure 3B. Higher values of sensitivities are observed for the proposed approach in case of HSP 20, HSP40, HSP90, and HSP100 whereas it is seen to be higher for PredHSP in other two families (Figure 3A). However, in terms of sensitivities, the developed approach outperformed iHSP-PseRAAAC for classification of all the HSP families (Figure 3A). Though, specificities for all the three approaches are observed at par (~97%) for HSP20, these are observed to be higher for the proposed approach in rest of the five families of HSPs. Except HSP40, MCC of the proposed computational method is also seen to be higher than that of both PredHSP and iHSP-PseRAAAC in rest of the families. Furthermore, average performance metrics of the proposed approach are not only seen to be higher than that of Ahmad et al. (2015) approach, but also over PredHSP and iHSP-PseRAAAC (Figure 3B). Since, datasets are highly imbalanced, values of AUC-PR are also computed. It is observed that except for HSP60, values of AUC-PR for the proposed approach are higher than that of PredHSP and iHSP-PseRAAAC in respect of classifying other families of HSPs (Table 5). Further, it is seen that except for HSP20, PredHSP outperformed iHSP-PseRAAAC in terms of AUC-PR measure (Table 5). Also, it is observed that the value of AUC-PR is lowest for prediction of HSP90 and highest for the prediction of HSP100.

**Table 5:** Values of AUC-PR for classification of different families of HSP, by the proposed approach and two other existing approaches.

**HSP family**	**Proposed**	**PredHSP**	**iHSP-PseRAAAC**
HSP20	0.4860	0.4418	0.4640
HSP40	0.4884	0.4703	0.4498
HSP60	0.4516	0.4629	0.4307
HSP70	0.4633	0.4419	0.4371
HSP90	0.4277	0.4101	0.3933
HSP100	0.4935	0.4880	0.4635

### Performance analysis using independent dataset

By using 2,181 HSPs and 2,181 non-HSPs (randomly drawn from available non-HSPs) as training dataset, prediction was made for the independent dataset consisting of 96 human and 55 rice HSPs. In human, 85 are correctly predicted by both the approaches whereas in rice 54 and 53 HSPs are correctly predicted by the proposed approach and PredHSP, respectively (Table 6). Further, out of 96 human HSPs, 84 and 83 are correctly predicted into their corresponding families, whereas in rice 53 and 52 HSPs are correctly predicted into their corresponding families by the proposed approach and PredHSP, respectively (Table 6). Besides independent set of HSPs, performances were also evaluated with an independent negative dataset containing 5,000 non-HSP sequences (as mentioned in “material and method” section). It is observed that the number of false positives predicted by PredHSP (1,398) are higher than that of proposed approach (810), where 560 predicted HSPs by the proposed approach are among the 1,398 of PredHSP (Figure 3C). So, it can be said that the proposed approach and PredHSP may be equally efficient in detecting the true positives, but number of false positives will be lesser for the proposed approach as compared to PredHSP.

**Table 6:** Number of observed and correctly predicted HSPs by the proposed approach and PredHSP for the independent dataset of 96 human and 55 rice HSPs.

**Dataset**	**HSP family**	**Observed**	**Predicted_family-wise**	
			**PredHSP**	**Proposed**
HGNC	HSP20	11	8 (2 non-HSP, 1 HSP40)	9 (2 non-HSP)
	HSP40	49	45 (4 non-HSP)	45 (4 non-HSP)
	HSP60	15	9 (5 non-HSP, 1 HSP70)	10 (4 non-HSPs, 1 HSP100)
	HSP70	17	17	17
	HSP90	4	4	3 (1 non-HSP)
RICE	HSP20^w^	14	12 (2 non-HSP)	13 (1 non-HSP)
	HSP60^w^	4	4	4
	HSP70^w^	7	7	7
	HSP90^w^	3	3	3
	HSP100^w^	3	3	3
	HSP70^s^	24	23 (1 HSP20)	23 (1 HSP20)

w *Wang et al. (2014) datset ^s^Sarkar et al. (2013)*.

### Performance analysis using interpro dataset

**Figure 4:**
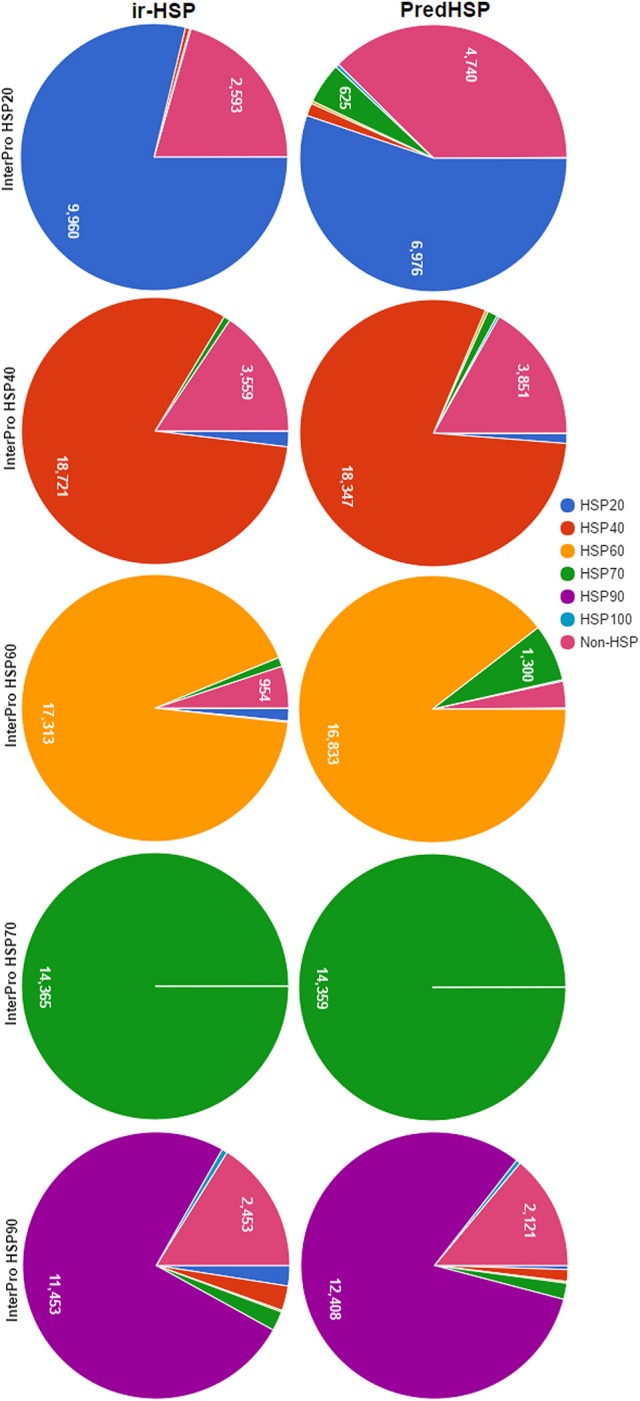
Pie charts showing the number of correctly predicted HSP by ir-HSP (proposed approach) and PredHSP, for different families of HSPs collected from InterPro database. In case of HSP20, HSP40, HSP60, number of correctly predicted HSPs by ir-HSP are higher than that of PredHSP, whereas in HSP90 PredHSP performed better than ir-HSP. Almost all the HSPs are correctly predicted by both the approaches for HSP70.

The same training dataset (2,181 HSPs and 2,181 non-HSPs) mentioned in the previous section was used for prediction of HSP sequences collected from InterPro. The sequences of the InterPro were also not present in the training dataset. Number of HSPs predicted into different families by the proposed approach (ir-HSP) and PredHSP are shown in Figure 4. It can be seen that the number of correctly predicted HSP20 (9,960), HSP40 (18,721), and HSP60 (17,313) by ir-HSP are higher than the correctly predicted HSP20 (6,976), HSP40 (18,347), and HSP60 (16,833) by PredHSP. On the contrary, number of correctly predicted HSP90 (12,408) by PredHSP are higher as compared to the number of HSP90 (11,453) correctly predicted by ir-HSP. Furthermore, it is observed that the total number of correctly identified HSPs by ir-HSP is higher than that of PredHSP. Specifically, out of 83,942 InterPro HSPs, number of correctly identified HSPs by ir-HSP and PredHSP are 74,383 and 72,622, respectively. Besides, almost all the HSP70 are seen to be correctly identified by both the methods.

### Prediction analysis of DnaJ proteins

Accuracies in predicting the DnaJ proteins by the proposed approach, JPred and JPPRED are given in Table 7. Both sensitivity and specificity of the developed approach are observed to be higher than that of JPred, for all the four types of J-Proteins. Though the sensitivities of JPPRED are seen to be higher, specificities are observed to be less than that of other two approaches. It is further observed that the sensitivity and specificity are more balanced in JPPRED as compared to the other two methods, and this may be due to the use of balanced dataset (number of observations in all the classes are almost same) in JPPRED that is obtained by employing synthetic minority over-sampling technique (SMOTE; Chawla et al., 2002). On the other hand, values of specificity for the proposed approach and JPred are observed to be higher than that of sensitivity due to imbalanced-ness (number of instances in Type-III is much higher than that of other classes). It is further observed that the overall accuracy (proportion of correctly predicted proteins for all the classes) of the proposed approach (94.7%) is at par with that of JPred (94.06%) but much higher than that of JPPRED (86.23%). Further, the proposed approach is observed to outperform JPred in terms of AUC-PR, as far as the classifications of four types of DnaJ proteins are concerned (Table 7). We have also tabulated all the methods along with their features that have been used for prediction of HSPs in earlier studies and the same is provided as Table S1 in Data Sheet 1.

**Table 7:** Performance metrics for the proposed approach, JPPRED and JPred with regard to classification of four types of DnaJ protein**s**.

**Method**	**J-Protein**	**Sensitivity**	**Specificity**	**Overall accuracy**	**AUC-ROC**	**AUC-PR**
Proposed	Type I	0.762	0.989	94.7	0.951	0.452
	Type II	0.547	0.990		0.853	0.374
	Type III	0.984	0.725		0.852	0.303
	Type IV	0.591	0.987		0.823	0.381
JPPRED^*^	Type I	0.921	0.859	86.23	–	–
	Type II	0.782	0.866			
	Type III	0.861	0.877			
	Type IV	1	0.86			
JPred	Type I	0.746	0.988	94.06	0.943	0.442
	Type II	0.491	0.991		0.824	0.353
	Type III	0.986	0.62		0.851	0.277
	Type IV	0.381	1		0.801	0.375

* *No source code or tool is available for running JPPRED. Besides, a combination of different features has been used in JPPRED which is not clear from the manuscript, and that is why the results could not be reproduced. Thus, we could not able to compute AUC-ROC and AUC-PR for JPPRED. The sensitivity, specificity and overall accuracy of JPPRED reported in the table are taken from the corresponding publication*.

### Analysis of proteome-wide prediction of HSPs

**Figure 5:**
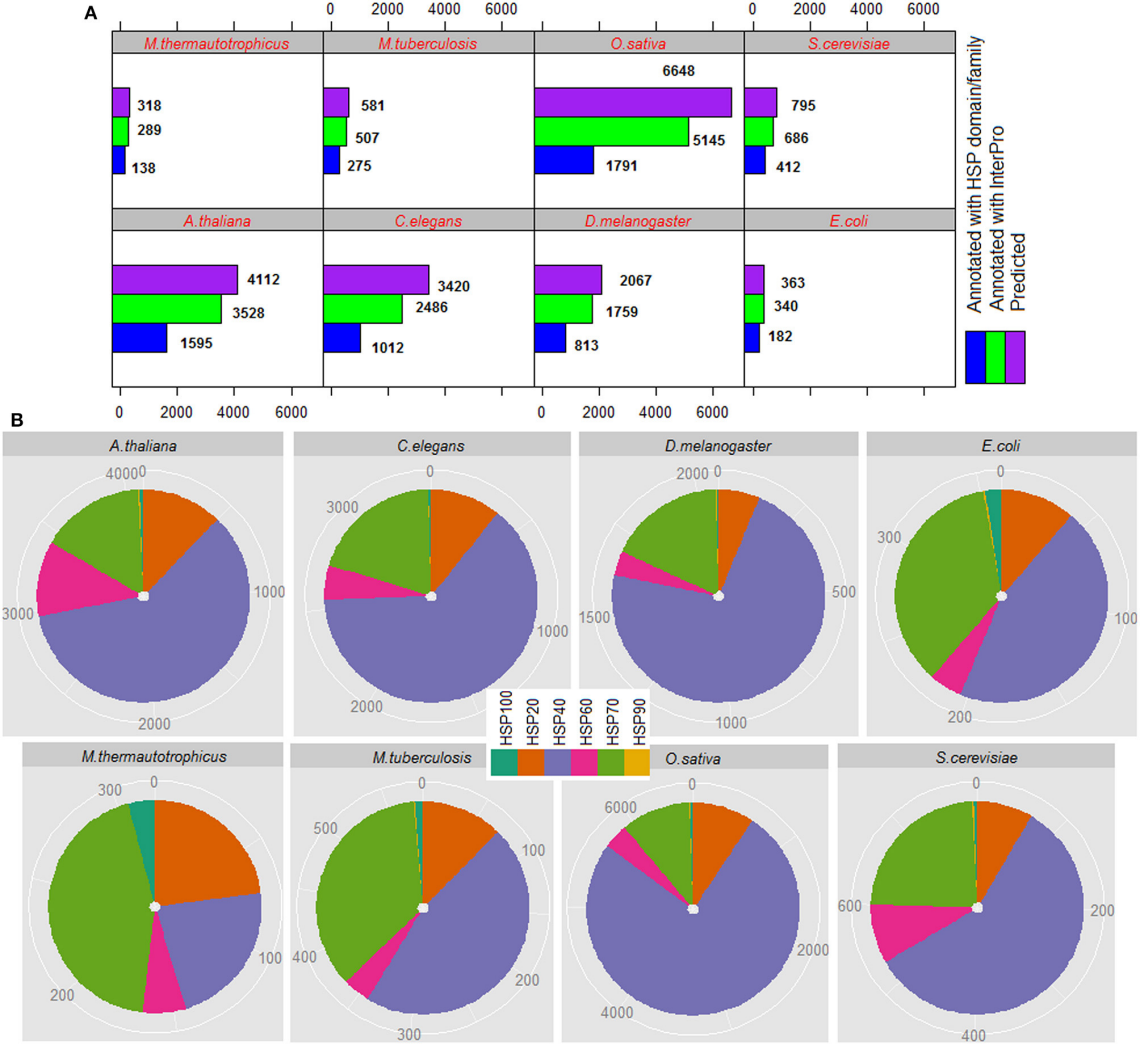
**(A)** Proteome-wide prediction of HSPs for eight different species, by using the proposed approach. It can be seen that ~50% of the predicted HSPs are annotated with InterPro HSP family/domain. **(B)** Family-wise distribution of predicted HSPs, where it is seen that >50% HSPs are predicted with HSP40 and <1% with HSP90.

The total number of HSPs predicted in each species, number of predicted HSPs annotated with InterPro and number of predicted HSPs annotated with HSP domains/families are shown in Figure 5A, and family-wise annotation of predicted HSPs are shown in Figure 5B. The proposed approach predicted 318 HSPs in *M. thermautotrophicus*, 362 in *E. coli*, 581 in *M. tuberculosis*, 795 in *S. cerevisiae*, 4,112 in *A. thaliana*, 6,648 in *O. sativa*, 3,420 in *C. elegans*, and 2,067 in *D. melanogaster*. It is seen that the percentage of HSPs are higher in both plant species (*A. thaliana* and *O. sativa*) than in other organisms. This could be due to the extra biotic and abiotic stress the plants tolerate due to their immobile nature (Al-Whaibi, 2011; Park and Seo, 2015). Further, highest number of HSPs are predicted with HSP40 followed by HSP70 and HSP20 whereas lowest number of HSPs are predicted with HSP90 (Figure 5B). In particular, >50% HSPs are predicted with HSP40 and <1% are predicted with HSP90. Out of total HSPs annotated with InterPro, ~50% of them are found to be annotated with HSP families/domains in each species (Figure 5A).

### Performance analysis using blast algorithms

To assess the performance using homology-based method, the most widely used Basic Local Alignment Search Tool (BLAST; Altschul et al., 1990) of NCBI was opted. Two different versions of protein blast i.e., Blastp and Delta-Blast with three different e-values i.e., 0.1, 0.01, and 0.001 were used for this purpose. Moreover, the classification of HSPs and non-HSP was made using 2,181 HSPs and 2,181 non-HSPs (as mentioned in section Performance Analysis using Independent Dataset) and performance was assessed through five-fold cross validation technique. For cross validation, the offline version of Blast software was installed in a local server, where Blastp and Delta-Blast algorithms were executed. In each fold of the cross validation, the training dataset was used as the database and the corresponding test set was used as query. Each query sequence was predicted as the HSP or non-HSP category based on the top hit found in the blast search. From the analysis it is seen that though the number of false positives are much less, no hits are found for many of the true positives. In particular, no hits are found for ~23, ~25, and ~26% of true HSPs with e-values i.e., 0.1, 0.01, and 0.001, respectively, in both Blastp and Delta-Blast. Thus, by using homology-based method there is a probability of losing information on true positives.

### Online prediction server: ir-HSP

A web server named as “ir-HSP” has been established and hosted at http://cabgrid.res.in:8080/ir-hsp to facilitate the prediction of HSPs up to the level of families and sub-types.

**Figure 6:**
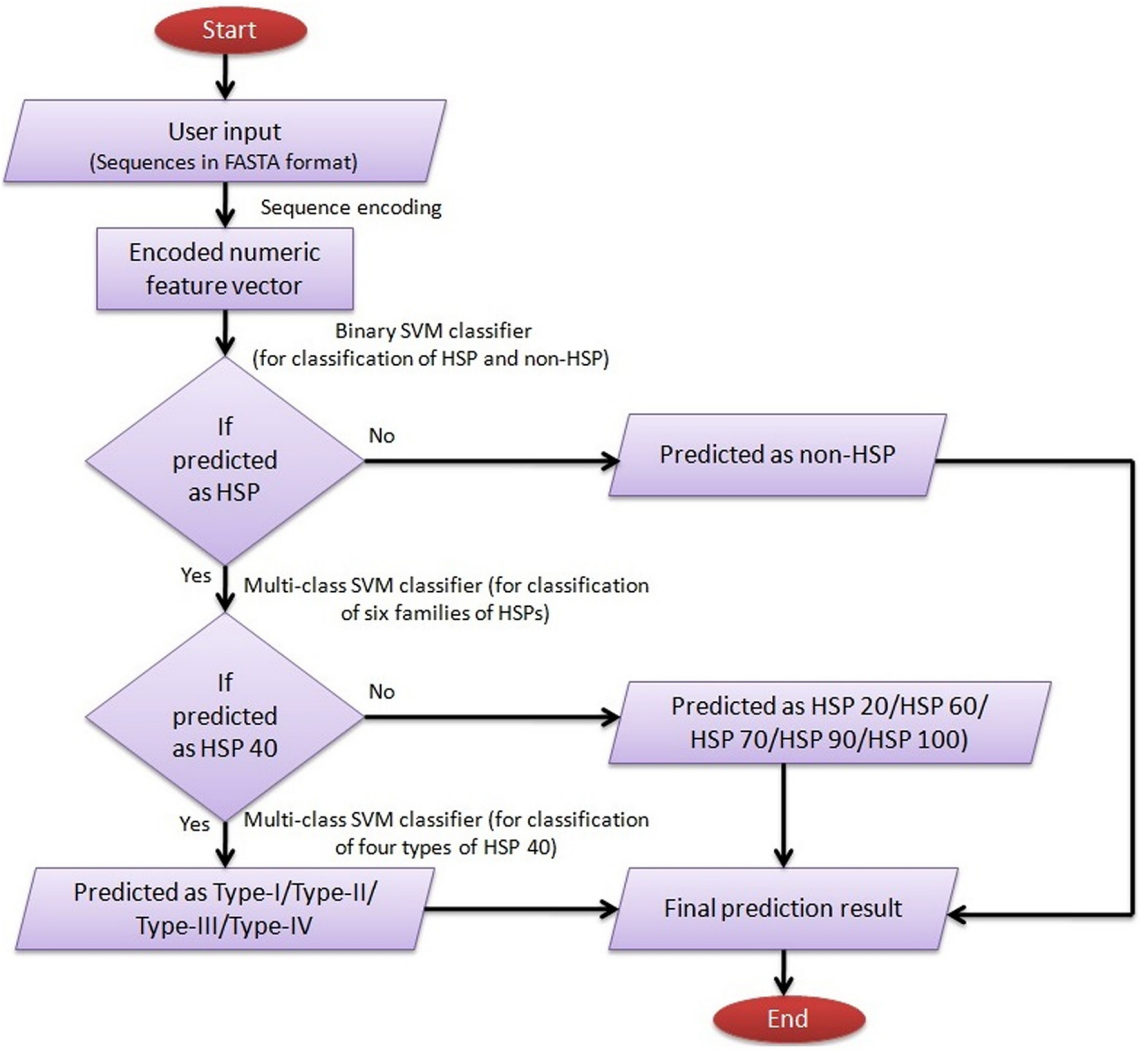
Flow diagram showing the step-wise working procedure of the developed prediction server ir-HSP.

For user guidance with regard to input-output, execution and interpretation of results, a help page has been provided in the main menu. The SVM architecture for predicting HSPs, their families and subtypes of DnaJ proteins by ir-HSP is explained through a flow diagram (Figure 6). The results are displayed in a tabular format with four columns. The first to fourth columns, respectively, represent the serial number, sequence identifier, types of predicted HSP (with sub-type of DnaJ, if predicted as HSP40) or non-HSP, and probabilities with which they are predicted in the respective classes. For reproducible research, links to download the datasets used to train the prediction server and other datasets used in this study are also provided at http://cabgrid.res.in:8080/ir-hsp/dataset.html.

## Discussion

The discovery of heat shock response by Ritossa (1962) in the salivary gland of drosophila larvae and subsequent recognition of HSPs have laid down the foundation for expanding research on HSPs (Tissieres et al., 1974; Morana et al., 1978). As molecular chaperones, HSPs are vital bio-molecules that play pivotal role in maintaining the structure of the cytoplasm in eukaryotes and safeguarding life against proteotoxic stress (Pratt and Toft, 1997; Csermely et al., 1998). Further, the broad range of functions of HSPs makes them an attractive target for pharmacological interventions. It is also believed that the diversity present in different families of HSP makes the plants to tolerate different biotic and abiotic stresses. Specifically, till date, 52 plant species including maize, soybean, cotton, potato have been genetically modified for heat stress tolerance (Al-Whaibi, 2011). Due to diversified nature of HSPs and wide range of functions, development of an automated method for timely and reliably predicting HSPs and their families is indispensable in the area of proteomics research (Feng et al., 2013). Keeping this in view, this study presents an automated system for identification of HSPs, their families and sub-types of DnaJ proteins in a single frame work as well as with higher accuracy.

In the proposed approach, the GPC features were used as input for prediction by employing SVM predictor. The accuracy under GPC feature set was found higher than that of DPC feature set used in PredHSP, which resembles with the finding of Brinda et al. (2015). Though it is true that the number of features in GPC-0123 feature set were higher than that of other feature sets, this may not be the only reason for getting higher accuracy because the number of features in ACF-2 were also higher than that of CTD but the accuracies were still lower than that of CTD feature set. Instead of using imbalanced dataset, performance of the proposed approach was assessed using balanced dataset to avoid biasness toward the major class (Chou, 2013; Chen et al., 2015). Moreover to assess the consistency with different non-HSP sequences, performance of the proposed approach was assessed over 100 sample sets, which seems to be more logical as compared to that of using one sample set in PredHSP (Kumar et al., 2016).

The DPC of standard amino acids was first used by Ahmad et al. (2015) for classification of six different families of HSPs, which was later adopted in PredHSP. This may be the possible reason that the overall sensitivity, specificity, accuracy and MCC were found to be approximately same for PredHSP and Ahmad et al. (2015) approach (Figure 3B). Though DPC feature set was initially used in iHSP-PseRAAAC, it was based on the reduced amino acid alphabet (Etchebest et al., 2007). However, GPC features were first time used in this study, and the accuracies under this feature set were found higher than that of PredHSP, iHSP-PseRAAAC, and Ahmad et al. (2015) approach with respect to classification of six different families of HSPs (Figure 3B). On the other hand, accuracy was found to be lowest for iHSP-PseRAAAC and this may be due to the use of reduced amino acid alphabet by which the variability present in the dataset was not captured well by the prediction model. In respect of predicting four types of DnaJ proteins, the proposed approach achieved high accuracy than that of JPred. Though the JPPRED achieved higher accuracy in terms of sensitivity, the developed approach outperformed JPPRED in terms of overall accuracy. Moreover, no computational tool is available for JPPRED to predict DnaJ proteins, which further limits its application with real-world protein sequence data. However, number of features used in JPPRED (224) is almost half of those used in the proposed approach (484) and JPpred (512). Nevertheless, it can be said that the proposed approach will supplement the existing approaches in predicting the four types of DnaJ proteins.

With the independent datasets of 96 human HSPs and 55 rice HSPs, almost same number of HSPs were correctly predicted into their corresponding families by both the proposed approach and PredHSP. However, the number of false positives were found higher for PredHSP as compared to the proposed approach. Furthermore, based on the InterPro dataset of HSP families/domain, the proposed approach was found to achieve higher accuracies for HSP20, HSP40, and HSP60 than that of PredHSP. On the other hand, PredHSP performed better than the proposed approach for HSP90. In case of HSP70, almost all the sequences were correctly predicted by both the methods and this may be due to the fact that Hsp70 proteins are highly conserved. In particular, HSP70 contains a conserved peptide binding domain, an ATPase domain, a region at the middle having protease sensitive sites and a C-terminal region enriched with G/P amino acids that enable the proteins to bind with co-chaperones and other HSPs (Hartl, 1996; Tavaria et al., 1996; Bukau et al., 2006; Daugaard et al., 2007).

The performance of the proposed approach was further assessed at proteome level by using 8 different proteome datasets. Though, most of the predicted HSP sequences were annotated with InterPro domain, ~50% of them were found to be annotated with HSP domains/families in each species. Further, most of them were found to be annotated with HSP40 followed by HSP20. In particular, number of predicted HSP40s were found to be higher for eukaryotes that resembles with earlier study (Wacker and Muchowski, 2006). Since ~50% of predicted HSPs were found to be annotated with HSP domains/families, the developed computational method is expected to supplement the existing approaches for sequence annotation at proteome level. The developed prediction server ir-HSP will be of great help for the experimental scientists to get the required results without going into mathematical details.

## Author contributions

PM and AR: conception and design of the work; PM, TS, and SG: acquisition, analysis, and interpretation of data; PM, SG, TS, and AR: drafting the manuscript; PM, AR, TS, and SG: revising the manuscript; All authors read and approved the final version of the manuscript.

### Conflict of interest statement

The authors declare that the research was conducted in the absence of any commercial or financial relationships that could be construed as a potential conflict of interest.

## Supplementary material

Data Sheet 1 A brief description about different feature selection techniques used in this study for selection of important features.
