# Supplementary material for: ir-HSP: Improved Recognition of Heat Shock Proteins, Their Families and Sub-types Based On g-Spaced Di-peptide Features and Support Vector Machine
Source: Front Genet. 2018 Jan 11;8:235. doi: 10.3389/fgene.2017.00235 (PMC5770798; doi:10.3389/fgene.2017.00235)
Supplement: Data Sheet 1 — A brief description about different feature selection techniques used in this study for selection of important features. [file DataSheet1.DOCX]

**A brief description about the feature selection techniques**

We employed five different feature selection techniques viz., F-measure (Golub et al., 1999), Information gain (Alhaj et al., 2016), LASSO (Tibshirani, 1996), Random Forest (Breiman, 2001) and SVM (Cortes and Vapnik, 1995) to select important features. In F-measure, the F-score was computed for each feature and the features with higher values of F-score were considered as important features. In case of information gain, each attribute is evaluated individually based on the information gain with respect to the class, where the attributes with higher values of information gain were considered more important. Based on SVM classifier, attributes were ranked by the square of the weight assigned by the SVM, where larger value indicates important attributes. By using Random forest methodology, importance of each variable was computed based on the mean decrease in accuracy of each attributes, and the variable with larger mean decrease in accuracy was considered more important as compared to others. In LASSO, features obtained with non-zero coefficient were considered important, where the features were ranked with the square of the values of coefficients.

**References**

Alhaj, T.A., Siraj, M.M., Zainal, A., Elshoush, H.T., and Elhaj, F. (2016). Feature selection using information gain for improved structural-based alert correlation. *PLoS One.* 11, e0166017.

Breiman, L. (2001). Random Forests. *Mach. Learn*. 45, 5–32.

Cortes, C., and Vapnik, V. (1995). Support-vector networks. *Mach. Learn*. 20, 273–297.

Golub, T., Slomin, D., Tamayo, P., Huard, C., Gaasenbeek, M., Mesirov, J., et al. (1999). Molecular classification of cancer: class discovery and class prediction by gene expression monitoring. *Science* 286, 531-537.

Tibshirani, R. (1996). Regression shrinkage and selection via the Lasso, *J. R. Stat. Soc. Ser. B.* 58, 267–288.

**Table S1.** A summary of the HSP prediction approaches.

| **Method** | **Features used** | **Input required** | **Purpose** |
| --- | --- | --- | --- |
| PredHSP | Di-peptide compositions | Protein sequences | Prediction of HSPs and their family types |
| iHSP-PseRAAAC | Pseudo amino compositions of reduced alphabets | Heat shock protein sequences | Prediction of six families of HSPs |
| Jpred | Di-peptide compositions of clustered amino acids | DnaJ protein sequences | Prediction of four types of DnaJ proteins |
| JPPRED | Combinations of pseudo amino acid compositions, split amino acid compositions and PSSM features | DnaJ protein sequences | Prediction of four types of DnaJ proteins |
| Ahmad et al. (2015) | Di-peptide compositions | Heat shock protein sequences | Prediction of HSP families and DnaJ proteins |
| Proposed (ir-HSP) | G-spaced di-peptide compositions | Protein sequences | Prediction of HSPs, their family types and four types of DnaJ proteins |
